# Efficacy of Corticosteroids for Sore Throat Management in Adults: A Systematic Review and Meta-Analysis of Randomized Trials

**DOI:** 10.7759/cureus.67740

**Published:** 2024-08-25

**Authors:** Saad Alqahtani, Tarfah Rashed I Alsharidi, Mohammed A Alelaiwi, Lama M Albelowi, Abdulaziz Saeed Alserhani, Ziyad A Alhosan, Rema M Alhazmi, Mohammed A Alaithan, Abdullah O Almutairi, Lama Alqahtani, Abdullah S Alhammad, Hotaf F Aljahdali, Faten Althomali, Maryam A Basalamah

**Affiliations:** 1 Consultant Family Medicine, King Salman Armed Forces Hospital, Tabuk, SAU; 2 Collage of Medicine, King Saud University, Riyadh, SAU; 3 Medicine and Surgery, Imam Muhammad Ibn Saud Islamic University, Riyadh, SAU; 4 College of Medicine, Taibah University, Madinah, SAU; 5 College of Medicine, King Khalid University Hospital, Abha, SAU; 6 College of Medicine, King Saud University, Riyadh, SAU; 7 Family Medicine, King Faisal University, Al-Ahsa, SAU; 8 College of Medicine, Unaizah College of Medicine and Medical Sciences, Qassim University, Al-Qassim, SAU; 9 College of Medicine, Alfaisal University, Riyadh, SAU; 10 College of Medicine, Qassim University, Riyadh, SAU; 11 Medicine, King Abdulaziz University, Jeddah, SAU; 12 Medicine, Taif University, Taif, SAU; 13 Medicine, Ibn Sina College, Jeddah, SAU

**Keywords:** meta-analysis, clinical trials, pharyngitis, sore throat, corticosteroids

## Abstract

Sore throat (acute pharyngitis) is among the most common complaints among adults and is a reason for seeking healthcare globally. Antibiotics are widely used among patients with infectious sore throat. Previous research has indicated that corticosteroids could offer an alternative symptomatic treatment for sore throats. To estimate the corticosteroid efficacy as an additional therapy for sore throat adult patients, the literature search included PubMed, Medline, OVID, Cochrane CENTRAL, and Scopus for articles published until July 1st, 2024. The outcomes included the onset of pain relief (average time), complete resolution of pain (average time), absolute reduction of pain at 24 and 48 hours, requirement of antibiotics, and adverse effects related to treatment. Standardized mean difference (SMD) and risk difference were used to report numerical and dichotomous results. Five studies were included. Among the five included studies, corticosteroids showed significant effectiveness in resolving pain at 24 hours (average risk difference: 0.2200, 95% CI: 0.0500 to 0.3899, p = 0.0112) but with notable heterogeneity (I² = 82.4255%). At 48 hours, the benefit was not statistically significant (average risk difference: 0.4063, 95% CI: -0.1857 to 0.9984, p = 0.1786, I² = 98.9219%). Corticosteroids also decreased the average time to onset of pain relief (average SMD: -0.6590, 95% CI: -1.2857 to -0.0323, p = 0.0393, I² = 89.7914%), although with high heterogeneity. Other findings indicated a possible reduction in antibiotic use and fewer days missed from work. Adverse effects were minimal and occurred at similar rates in both corticosteroid and placebo arms. Corticosteroids can decrease pain intensity and duration in adults with acute sore throats. However, significant heterogeneity among studies and methodological limitations render the overall evidence inconclusive. While some studies noted reduced antibiotic use and lower symptom recurrence, high-quality RCTs are needed to address these limitations and provide more definitive guidelines for corticosteroid use in treating acute pharyngitis.

## Introduction and background

Complaints among adults are a reason for seeking healthcare globally [1]. Viral infections account for around 85% of cases, while bacterial, fungal, smoking, intubation, and yelling account for 15% of sore throat causes [2]. Even though sore throat cases are primarily due to viral infections, they are considered one of the most common cases where using antibiotics is prevalent worldwide [3]. Sore throat management depends on using analgesics such as acetaminophen and non-steroidal anti-inflammatory drugs (NSAIDs) [4]. Although these medications have detectable safety profiles, a significant number of preventable adverse drug reactions (ADRs) associated with analgesics are still present [5].

Antibiotics could also manage a sore throat caused by bacterial infections such as streptococcal pharyngitis [6]. As it is commonly encountered in primary care, patients in outpatient visits are often treated with antibiotics [7]. Even though antibiotics are beneficial for treating sore throats with bacterial etiology, they present a risk for emerging cross‐resistance in several bacterial respiratory pathogenic bacteria [8]. However, physicians frequently prescribe antibiotics as they consider patients seeking care need this antibiotic course, while, in fact, patients mostly require pain relief [9].

Corticosteroids could offer an alternative symptomatic treatment for sore throats. A previous study found they reduce discomfort and duration until symptom improvement in patients with sore throats, especially those with severe exudative pharyngitis [10]. Another systematic review found that corticosteroids effectively relieved pain in acute pharyngitis. Despite the absence of severe adverse effects, the benefits of their use should be balanced with possible adverse drug effects [11].

While previous research has investigated the efficacy of corticosteroids across various age groups and populations, a detailed pooled analysis is still needed among adult populations. Therefore, we developed this review to evaluate the efficacy of corticosteroids as an additional treatment approach for infectious sore throat.

This systematic review and meta-analysis aim to address whether corticosteroids are effective in adults with sore throats in terms of reducing time to pain relief, achieving complete resolution of symptoms within 24 and 48 hours, and the occurrence of adverse events in comparison to placebo or standard of care for randomized controlled trial (RCT) study. Our findings will contribute to the existing literature and guide researchers and practitioners in advancing the understanding of corticosteroid use in treating pharyngitis.

## Review

Methods 

Methodology

The Preferred Reporting Items for Systematic Reviews and Meta-Analyses (PRISMA) criteria were followed in the reporting of the study, which was carried out following the Cochrane Handbook for Systematic Reviews and meta-analysis guidelines [12].

Eligibility Criteria

The review included RCTs published in English without time constraints.

Participants

The studies involved adult patients exhibiting signs of tonsillitis, pharyngitis, or sore throat and were conducted in both emergency and primary care environments.

Intervention

Comparison between corticosteroids and standard clinical care.

Exclusion Criteria

The excluded studies were (1) studies with no full-access link, (2) study types such as case reports, letters, observational studies, review articles, and systematic review articles, (3) duplicate studies found in multiple databases or sources, (4) studies of subjects who were hospitalized, (5) studies of patients who are having kissing disease, sore throat after surgery or postoperative sore throat, heartburn, or peritonsillar abscess, and (6) studies that included children.

Search Strategy

The search used five databases: Pubmed, Medline, OVID, Cochrane CENTRAL, and Scopus. We conducted our search without applying any filters, covering the period from the inception of the database until July 1, 2024. Key terms for sore throat conditions included “nasopharyngitis, tonsillitis, pharyngitis, nasopharyngi, or Streptococcus.” We targeted studies using steroids, glucocorticoids, hydroxycorticosteroids, hydroxypregnenolone, corticosterone, paramethasone, or betamethasone. We focused on studies designed as randomized trials. The combined search string was `[[[[nasopharyng] OR [Tonsillitis] OR [Pharyngitis] OR [nasopharyngi] OR [Streptococcus]] AND [[steroid] OR [Glucocorticoids] OR [Hydroxycorticosteroid] OR [hydroxypregnenolone] OR [corticosterone] OR [paramethasone] OR [betamethasone]]] AND [[clinical trial] OR [RCT] OR [randomized clinical trial]]]]`(see Table 3 of Appendix A).

Studies Selection

Two independent reviewers performed the online search, screened the titles and abstracts, and examined the full text of relevant articles. Any disagreements were resolved by consensus.

Data Extraction

We collected the following data: authors' name, year, objective, methodology, pathogen positive (%), steroid type, dose and duration, antibiotic use (%), analgesic use (%), and outcomes regarding participants' complete resolution of pain on different time points; average time to initial pain relief; the average time to resolution of pain; overall reduction of pain at 24 hours; duration of non-tolerable symptoms; antibiotics requirement; and number of adverse events caused by treatment.

Measured Outcome

Primary outcomes: Mean risk difference of resolution of pain, average time to initial pain relief, average time to overall pain resolution, and overall pain reduction at 24 hours.

Secondary outcome: Incidence of adverse events caused by the treatment.

Assessment of the Risk of Bias in the Included Studies

We evaluated the risk of bias (ROB) utilizing the ROB2 tool for randomized clinical trials (RCTs) [13]. The ROB2 tool includes five domains: randomization, deviations from the assigned treatment, missing data, measurement of the outcome, and selective reporting of the outcomes and results. Then, the overall ROB was addressed by selecting the highest level of ROB from these five domains.

Data Synthesis

Meta-analysis was conducted by using the Jamovi Software (version 2.3) [14]. Numerical outcomes were analyzed. The difference was presented using the standardized mean difference (SMD). For dichotomous outcomes, we calculated the risk difference. We assessed and identified significant heterogeneity when the I2 index was 50% or higher. If significant heterogeneity was found, the studies’ findings were pooled using the DerSimonian-Laird random effects model. Statistical significance for comparisons between the two groups was determined using a p-value of less than 0.05. We did not evaluate publication bias because there were fewer than 10 eligible studies.

Results 

**Figure 1:**
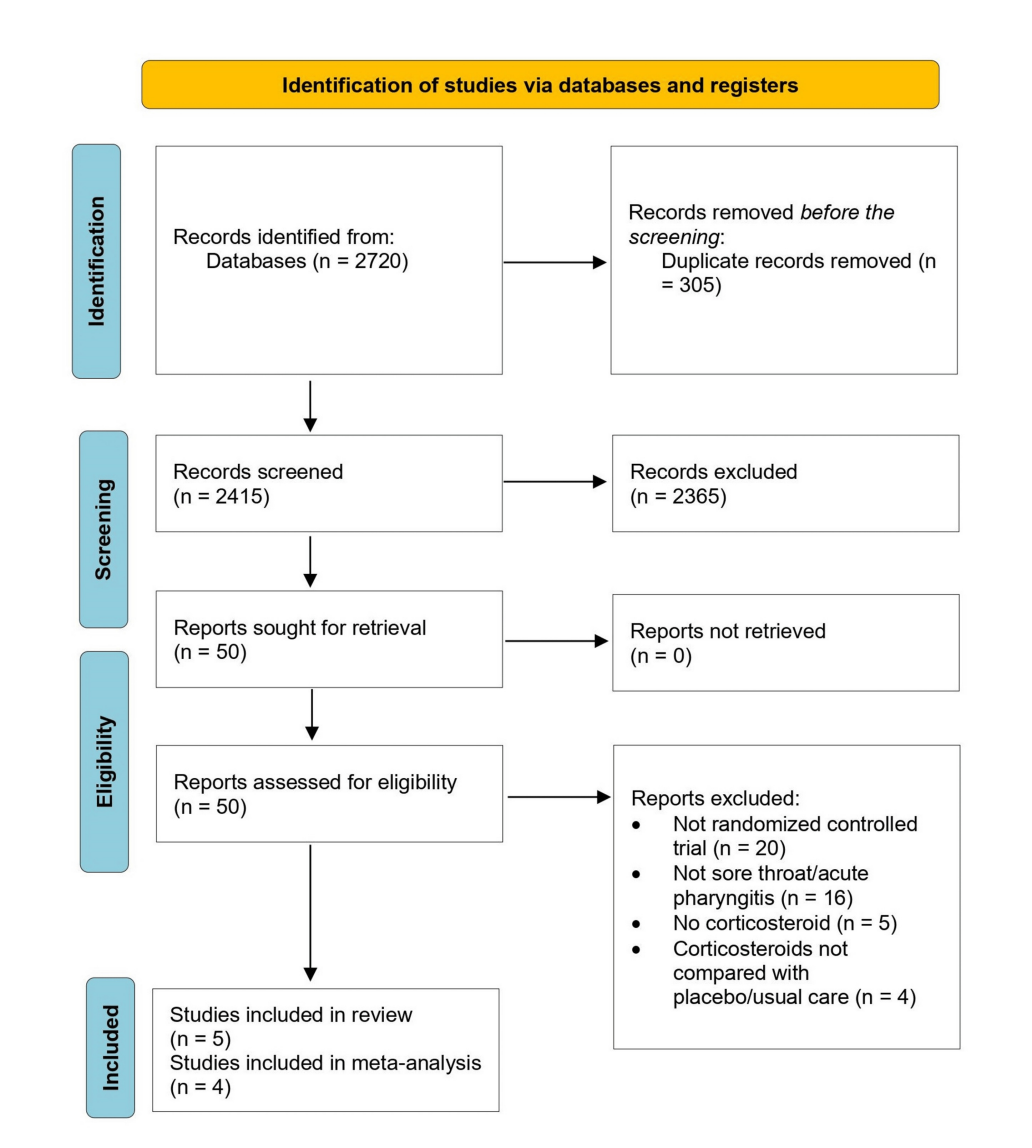
Selection of studies in the review of corticosteroids for the treatment of sore throat in adults

Through the systematic literature search, we identified 2415 titles and abstracts, of which 50 were selected for retrieval. Of the 50 studies, 45 were excluded (20 were not randomized trials; 16 had no individuals with a sore throat; in 9 studies, corticosteroids were not mentioned in the studies as an intervention or compared with a placebo/usual care). Figure 1 illustrates the details of the study selection process. Finally, the review included five studies that were eligible and enrolled 866 participants [15-19]. Three studies included patients from emergency departments (ED) and two from primary care [15-19]. All studies enrolled adults. The most prevalent intervention in three studies was a single 10 mg dose of oral dexamethasone for adults. In two studies, all patients were treated with antibiotics and analgesics as part of their standard care [16,18]. In one trial, all patients were given antibiotics, and analgesics were administered based on the physician’s judgment [19]. In two trials, patients in the usual care group were given antibiotics or analgesics based on the physician's judgment [15,17]. Table 1 presents the main characteristics of the included studies. 

**Table 1:** Characteristics of the included trials on corticosteroids for the management of sore throat A positive result on culture or rapid test for group A β hemolytic streptococcus (GABHS). ED, emergency department; NA, not available

Study	Setting	Population	Mean age (years)	Intervention (N)/control	Pathogen positive (%)	Steroid type	Dose and duration	Antibiotic use (%)		Analgesic use (%)	
								Intervention	Control	Intervention	Control
Hayward GN et al., 2017 [15]	Primary care	Adults	34	293/283	14.9	Dexamethasone (oral)	10 mg, single dose	39.9	39.0	77.1	78.9
Tasar A et al., 2008 [16]	ED	Adults	31.3	31/42	NR	Dexamethasone (IM)	8 mg, single dose	100	100	100	100
Kiderman A et al., 2005 [17]	Primary care	Adults	33.9	40/39	57.5	Prednisone (oral)	60 mg, single dose (100%) or for two days (50%)	51.4	63.2	NA	NA
Wei JL et al., 2002 [18]	ED	Adults	28.1	42/38	39.0	Dexamethasone (oral and IM)	10 mg, single dose	100	100	100	100
Marvez‐Valls EG et al., 2008 [19]	ED	Adults	29.2	31/27	NA	Dexamethasone (IM)	10 mg, single dose	100	100	NA	NA

**Figure 2:**
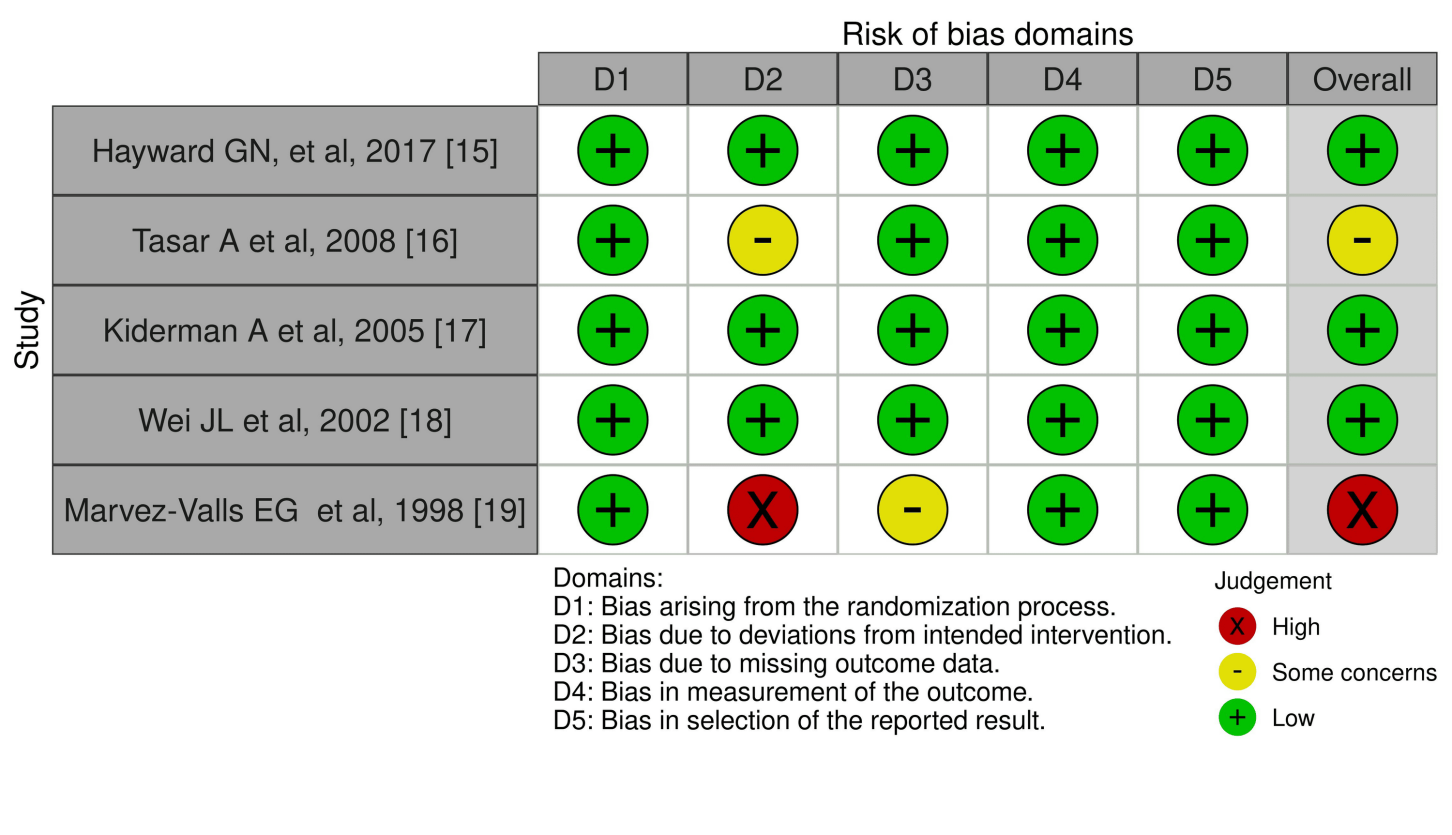
Traffic light plot

**Figure 3:**
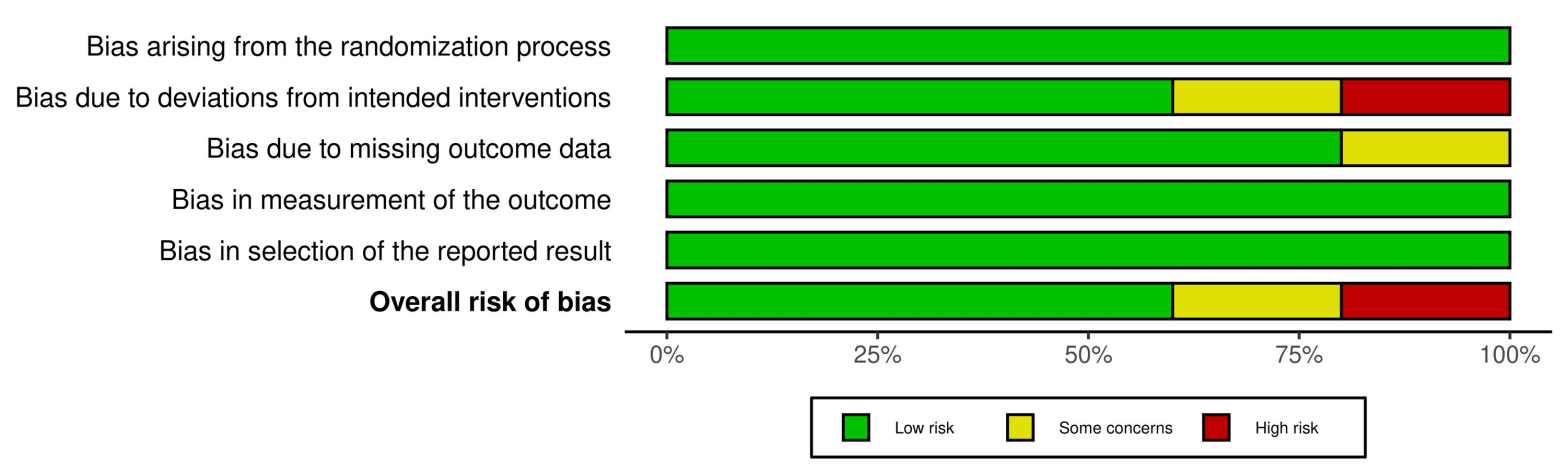
Summary of the overall ROB ROB, risk of bias

In our included studies, one RCT was at high ROB, and one study had some concerns [19]. The other three studies exhibited a low ROB across all domains. Figures were formatted using the Robvis visualization tool [20]. Figures 2, 3 show the summaries of the overall ROB.

Pain 

**Figure 4:**
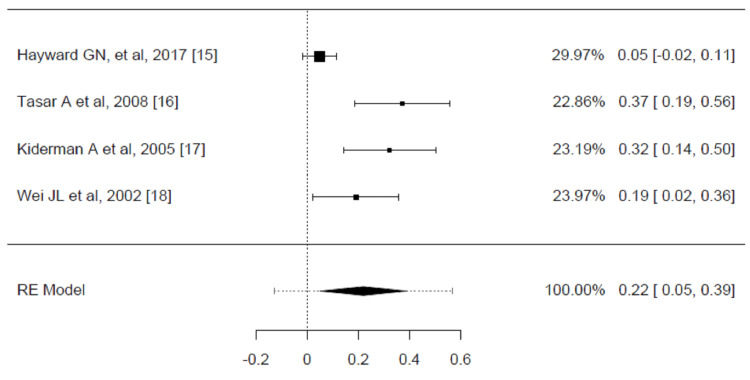
Risk difference for complete resolution of pain at 24 hours for corticosteroid vs. placebo groups in the review of the treatment of sore throat Pooled analysis calculated by DerSimonian-Laird random effects model.

Four RCTs documented full symptom resolution within 24 hours [15,16,17,18]. The estimated average risk difference was 0.2200 (95% CI: 0.0500 to 0.3899, p = 0.0112), which shows that corticosteroids are more effective than placebo in resolving pain at 24 hours. However, according to the Q-test, the true outcomes appear heterogeneous (p = 0.0007, I² = 82.4255%) (Figure 4).

**Figure 5:**
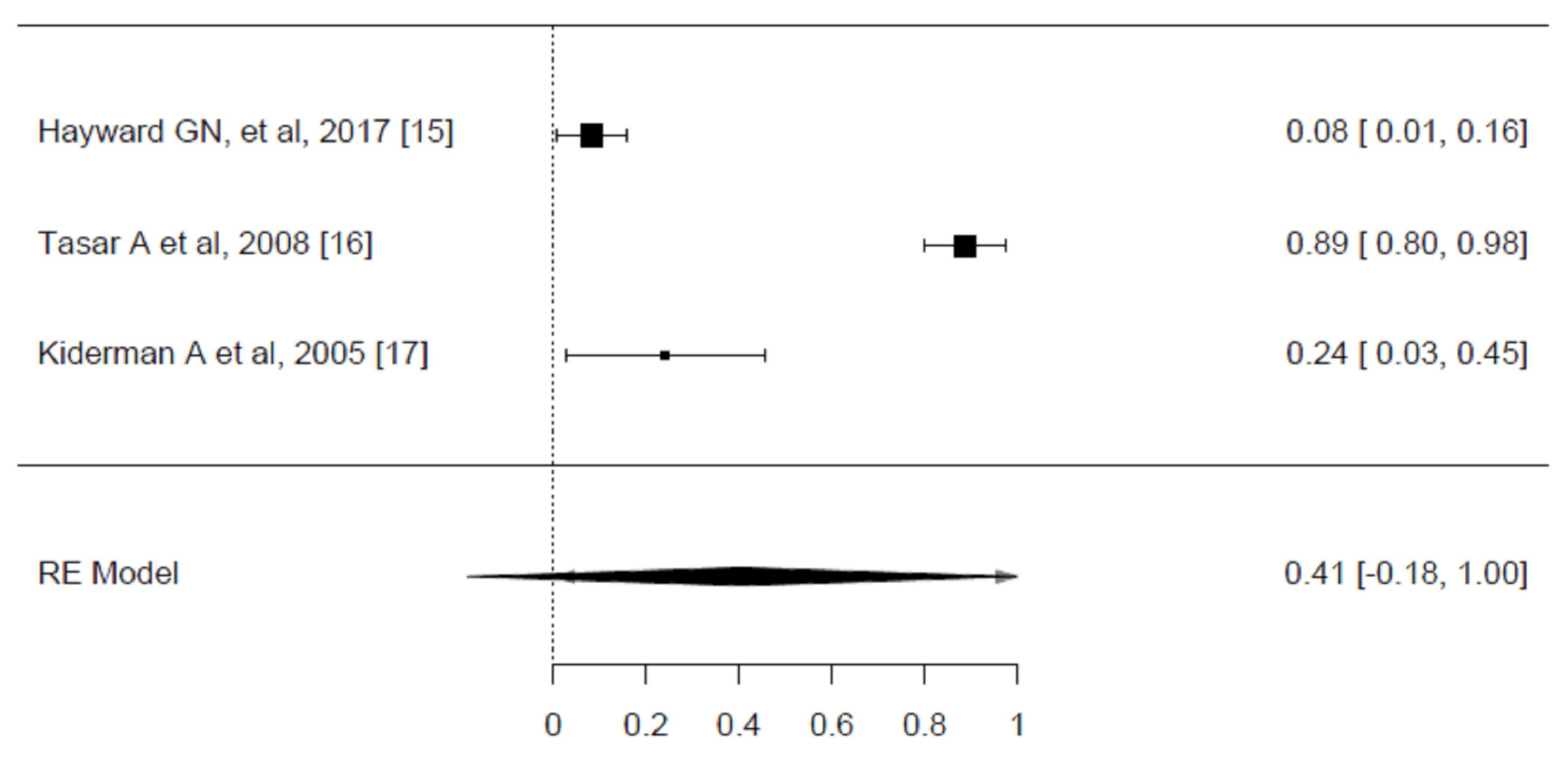
Risk difference for complete resolution of pain at 48 hours for corticosteroid vs. placebo groups in the review of the treatment of sore throat

A total of three trials were selected to be included in the analysis. These studies indicated that pain was completely resolved within 48 hours [15-17]. The estimated average risk difference based on the random effects model was 0.4063 (95% CI: -0.1857 to 0.9984, p = 0.1786). Based on the Q-test, the true outcomes show heterogeneity (p < 0.0001, I² = 98.9219%). The pooled analysis was measured using the DerSimonian-Laird random effects model (Figure 5).

**Figure 6:**
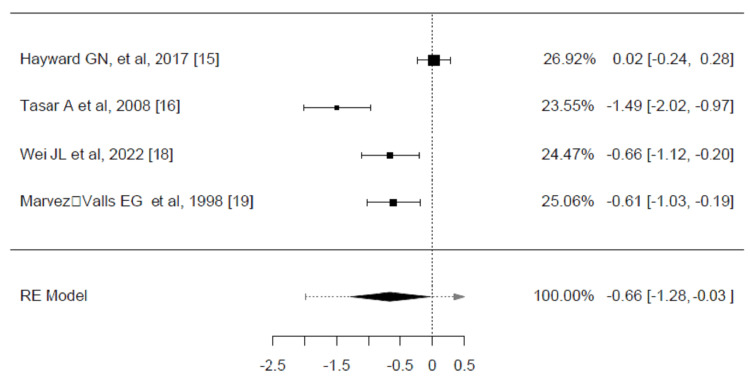
The weighted mean difference in the mean time to onset of pain relief (hours) between corticosteroids and placebo groups in the review of the treatment of sore throat Pooled analysis calculated by DerSimonian-Laird random effects model.

Four trials reported the average time to initial pain relief (hours), which were included in the analysis. The observed SMDs ranged from -1.4927 to 0.0281, with most estimates being negative (75%). The average SMD estimated was -0.6590 (95% CI: -1.2857 to -0.0323, p = 0.0393). Despite the significant difference, according to the Q-test, the true outcomes appear heterogeneous (p < 0.0001, I² = 89.7914%) (Figure 6).

**Figure 7:**
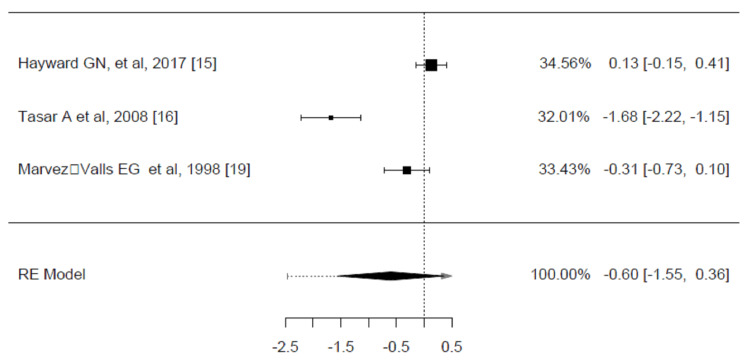
Weighted mean difference in the mean time to complete resolution of pain (hours) between corticosteroids and placebo groups in the review of the treatment of sore throat Pooled analysis calculated by DerSimonian-Laird random effects model.

A total of three trials were included in the analysis of the mean difference in the average time to complete the resolution of pain between corticosteroids and placebo groups [15,16,19]. The average SMD estimated was -0.5984 (95% CI: -1.5543 to 0.3576, p = 0.2199). Based on the Q-test, the true outcomes show heterogeneity (p < 0.0001, I² = 94.1780%) (Figure 7).

**Figure 8:**
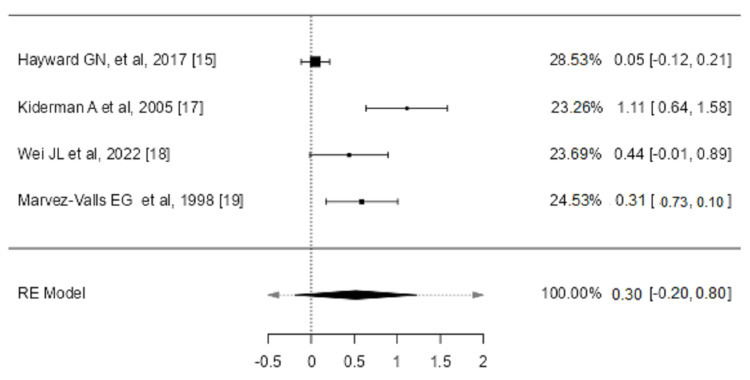
Weighted mean difference in an absolute reduction of pain at 24 hours (0-10; 0 = no pain, 10 = maximum pain) between corticosteroids and placebo groups in the review of the treatment of sore throat

We included four studies in the analysis to assess the weighted mean difference in overall reduction of pain at 24 hours among corticosteroids and placebo arms [15,17,18,19]. The average SMD estimated using the random effects model was 0.2990 (95% CI: -0.1995 to 0.7974, p = 0.2398). Based on the Q-test, the true outcomes show heterogeneity (p < 0.0001, I² = 87.3284%) (Figure 8).

Despite the significant findings revealed in some of the reported outcomes of pain, such as complete absence or cessation of pain at 24 and 48 hours and the time to initial pain relief, the results were inconclusive due to the heterogeneity evident by the meta-analysis of the included studies.

Other Outcomes 

One study's authors reported a possible reduction in antibiotic receipt in patients managed with corticosteroids [15]. In addition, Kiderman A et al. recommended a lower risk of recurrence or relapse of the symptoms [17]. Researchers noted that 55% of individuals managed by corticosteroids and 69% of those given a placebo took a leave of absence from work due to a sore throat, with a relative risk of 0.8.

Marvez-Valls et al. found that adults managed with corticosteroids missed an average of 0.4 days (SD 1.4). In contrast, those in the placebo group missed about 0.7 days on average (SD 1.4), resulting in a mean difference of -0.3 days [19]. None of the trials included in the study reported the duration of intolerable symptoms.

Except for one study, all included studies collected data on adverse effects through various methods, such as attendance or telephone contact (one study) and open-ended questions or diaries for self-reported adverse events (three trials). Table 2 outlines the adverse effects evaluated and the methods employed. Three studies found no adverse effects, whereas two reported adverse events in both the corticosteroid and control groups, potentially due to disease-related complications occurring at comparable rates [15-19]. Hayward et al. reported two serious adverse effects (hospitalizations due to ear, nose, and throat conditions) in the corticosteroid group (0.7%) and three such incidents in the placebo group (1.1%).

**Table 2:** Summary of adverse event assessments among trials included in the systematic review of corticosteroids for the treatment of sore throat Reflect investigators’ attempts not only to detect detrimental effects attributable to steroids but also treatment failures, relapses, and complications related to the disease.

Study	Methods used to assess adverse effects	Adverse effects assessed	Adverse effects reported
Hayward GN, et al., 2017 [15]	Attendance or telephone contact at any healthcare facility with symptoms or complications associated with a sore throat (defined as direct suppurative complications or presentation with symptoms of sore throat)	Any detrimental effect	Two severe adverse events (hospitalizations for conditions like pharyngeal or peritonsillar abscess, tonsillitis, and pneumonia) were observed in the corticosteroids group (0.7%), compared to three occurrences in the placebo group (1.1%)
Tasar A et al, 2008 [16]	Self-reported side effects at follow-up call	Complications associated with dexamethasone and azithromycin	NA
Kiderman A et al, 2005 [17]	NA	Any detrimental effect	NA
Wei JL et al, 2002 [18]	Self-reported side effects at follow-up call	Any detrimental effect	1 patient who received corticosteroids (3%) mentioned hiccups
Marvez‐Valls EG et al, 1998 [19]	Self-reported side effects at follow-up call	Any detrimental effect	NA

Discussion 

This review assessed the effects of corticosteroids on patients with acute sore throats. The results indicated that a single or double low dose of corticosteroids decreases the intensity and duration of pain, pain scores at 24 hours, and the time required for overall pain relief at both 24 and 48 hours. Consistent results were observed across studies for all pain-related outcomes, with the intervention group showing notable improvements in the time to pain alleviation. However, due to significant variability among the studies, the meta-analysis could not provide definitive conclusions for all outcomes.

Dexamethasone, commonly used in both oral and IM forms, was administered at a single dose of 10 mg in most studies. The exception is Kiderman A et al., 2005, who studied the effect of oral prednisone [17]. This could provide an advantage toward dexamethasone based on its efficacy in both oral and IM forms. The choice of dosage and duration did not indicate significant differences in outcomes, highlighting that both routes are viable based on clinical circumstances.

The decision to select oral or IM administration of steroids should consider the clinical setting, patient preferences, and the severity of symptoms. IM administration might be preferred in acute settings like the ED for its rapid onset of action, while oral administration could be more convenient in primary care with similar efficacy. The reviewed studies suggest that both routes are effective, and the decision should be customized to each patient's needs.

Among the included studies, Hayward GN et al. found that the major outcome of the study was the complete alleviation of sore throat infection 24 hours and 48 hours after taking a single oral dose of dexamethasone without administering immediate antibiotics, compared to a placebo [15]. The study found that while there was no significant difference in complete symptom resolution at 24 hours between the dexamethasone and placebo groups, significantly more dexamethasone groups experienced complete resolution at 48 hours than the placebo group. Similarly, other studies conducted on patients undergoing general anesthesia with endotracheal intubation found that administering dexamethasone preventively significantly decreased the frequency and intensity of postoperative sore throat compared to a placebo. In addition, Hansen B found that despite dexamethasone not significantly increasing complete symptom resolution at 24 hours compared to placebo, it resulted in significantly more participants experiencing complete resolution of sore throat symptoms at 48 hours [21,22]. Furthermore, in 2020, a systematic review and meta-analysis were conducted on nine studies involving children and adults suffering from sore throats who were administered oral or intramuscular corticosteroids in addition to antibiotics. The authors concluded that the addition of corticosteroids moderately increased both the resolution and improvement of pain [23].

Additionally, in 2005, a study found that adjuvant therapy with short-acting oral prednisone was both safe and more effective than placebo for relieving symptoms of acute pharyngitis in adults in general practice settings. Patients who received prednisone experienced quicker relief from throat pain, with no notable rise in bacteriological or clinical recurrences. The authors highlighted the benefits of steroid use in easing acute pharyngitis symptoms despite the minimal risks associated with the therapy [17]. This was consistent with another finding conducted in a systematic review and meta-analysis, which revealed that steroids could help manage acute pharyngitis. Still, there is insufficient evidence to endorse routine use [11].

According to Marvez-Valls et al., patients receiving betamethasone as an adjunct therapy for acute exudative pharyngitis experienced more frequent and quicker pain relief. The study found a notable reduction in pain scores at both the first (p = 0.006) and second (p = 0.02) follow-up visits among those patients with a positive culture for streptococcus species [19]. Another descriptive study conducted in the United Arab Emirates (UAE) found that dexamethasone was both safe and effective as an adjunct treatment for pain associated with acute β-hemolytic Streptococcal (GABHS) pharyngitis. The study indicated that nearly all patients experienced notable pain relief within 36 to 48 hours, with no reported side effects from dexamethasone [24].

Furthermore, it was found that single-dose (8 mg) intramuscular dexamethasone therapy added to the standard treatment resulted in faster pain relief in adults with acute exudative pharyngitis who exhibit three or four Centor criteria [16]. This was confirmed by a recent retrospective study among patients who were orally or intramuscularly administered 10 mg of dexamethasone as an adjuvant therapy with conventional treatments. The finding of this study showed that single-dose dexamethasone as an additional therapy with standard care was an effective and safe approach for patients having acute sore throats in primary care clinics [25]. An older study also proved this included in this review, which also revealed that the identification of a bacterial pathogen had a significant impact on the response to dexamethasone [18].

Limitations 

This review analyzed the efficacy of corticosteroid treatment among adult patients with pharyngitis. However, several limitations were evident regarding the RCTs' findings and study design. For instance, the self-reporting of VAS pain scores and having patients evaluate their pain resulted in a possible recall bias. Additionally, recording the total number of doses and the strength of analgesics taken by patients in each group was challenging. Moreover, many recruited participants dropped out. Missing patients’ cultures and controlling for confounding factors due to analgesic use were among the issues found in the included studies.

Implications

This systematic review and meta-analysis support the use of corticosteroids in the treatment of sore throats in adults by demonstrating faster pain relief and reduced pain intensity in the first 24 hours. However, heterogeneous results among the studies included suggest that these results need to be interpreted with caution. The variability in study design, patient populations, and corticosteroid regimens adds up to the inconclusiveness of the evidence. While corticosteroids could possibly present an alternative to classic analgesia and reduce antibiotic use, the evidence currently cannot support widespread adoption until more high-quality RCTs are conducted. Such future studies should look to address the main methodological limitations brought out in the review through a proper standardization of dosing and patient selection criteria so that clearer clinical practice guidelines would result. The low rate of reported adverse effects also suggests that corticosteroids could be a safe additional treatment, but little is known about their long-term safety in this respect. This review highlights the need for further research to firmly establish corticosteroids' role in sore throat management and the benefit-to-risk balance in such treatments. 

## Conclusions

This review assessed the efficacy of corticosteroid treatment among adult patients with pharyngitis. The findings recommend that corticosteroids can reduce the intensity and length of pain associated with acute sore throats among adult patients. Despite these findings, significant heterogeneity among the included studies indicated outcome variability. Furthermore, while some studies reported additional benefits, such as a reduced likelihood of antibiotic use and lower risk of symptom recurrence, the overall evidence remains inconclusive due to methodological limitations. Thus, high-quality RCTs are needed. These trials should address the existing limitations and provide more definitive evidence regarding corticosteroid use and guidelines in treating acute pharyngitis among adult patients. Future research should focus on optimizing study designs and standardizing outcome measures to ensure more reliable and generalizable results. Therefore, more studies on the possible beneficial and adverse events of steroids as adjunctive therapy to antibiotics in pharyngitis are essential.

## Appendices

**Table 3:** MESH terms and search strategy results

Database		Search strategy	Results
A)	PubMed	("nasopharyng"[Title/Abstract] OR "Tonsillitis"[Title/Abstract] OR "Pharyngitis"[Title/Abstract] OR "Streptococcus"[Title/Abstract]) AND ("steroid"[Title/Abstract] OR"Glucocorticoids"[Title/Abstract] OR "Hydroxycorticosteroid"[Title/Abstract] OR "hydroxypregnenolone"[Title/Abstract] OR "corticosterone"[Title/Abstract] OR "paramethasone"[Title/Abstract] OR "betamethasone"[Title/Abstract])	392
B)	Medline	1) ((((AB=(nasopharyng)) OR AB=(Tonsillitis))) OR TI=(Pharyngitis)) OR AB=(Streptococcus) 2) (((((AB=(steroid)) OR AB=(Glucocorticoids)) OR AB=(Hydroxycorticosteroid)) OR AB=(hydroxypregnenolone)) OR TI=(corticosterone)) OR AB=(paramethasone) #2 AND #1	523
C)	OVID	1) nasopharyng.m_titl, 2)Tonsillitis.mp. [mp=title, abstract, full text, caption text]. 3) Pharyngitis.[mp=title, abstract, full text, caption text]., 4) Streptococcus. [mp=title, abstract, full text, caption text]. 5) 1 or 2 or 3 or 4 6) steroid. [mp=title, abstract, full text, caption text]., 7) Glucocorticoids. [mp=title, abstract, full text, caption text], 8) Hydroxycorticosteroid. [mp=title, abstract, full text, caption text], 9) corticosterone.[mp=title, abstract, full text, caption text]., 10) paramethasone. [mp=title, abstract, full text, caption text],11) betamethasone [mp=title, abstract, full text, caption text] 12) 6 or 7 or 8 or 9 or 10 or 11 5 and 12	558
D)	Cochrane CENTRAL	1) “Tonsilitis AND” “Steroid” [Abstract], 2) “pharyngitis” AND “Steroid” [Abstract], 3) “Streptococcus” AND “Steroid”[Abstract]	134
E)	SCOPUS	1) TITLE-ABS ( tonsilitis ) AND TITLE-ABS ( steroid ), 2) TITLE-ABS (pharyngitis) AND TITLE-ABS (steroid ), 3) TITLE-ABS ( streptococcus ) AND TITLE-ABS ( steroid), 4) TITLE ( nasopharynx ) AND TITLE-ABS ( steroid ), 5) TITLE ( tonsillitis ) AND TITLE-ABS ( glucocorticoids ), 6) TITLE (pharyngitis) AND TITLE-ABS ( glucocorticoids ).	1113
